# Outpatient pediatric care during the COVID-19 pandemic, Almaty, Kazakhstan 2021–2022

**DOI:** 10.3389/fpubh.2025.1665990

**Published:** 2025-10-06

**Authors:** Nailya Kozhekenova, Milena Santric-Milicevic, Zhansaya Nurgaliyeva, Ainash Oshibayeva, Danilo Jeremic, Milan Dinic, Saltanat Kyrykbayeva, Zhanar Zhagiparova, Arshat Smasheva, Anastassiya Miller, Shyryn Tolekova, Natalya Glushkova

**Affiliations:** ^1^Faculty of Medicine and Health Care, Al-Farabi Kazakh National University, Almaty, Kazakhstan; ^2^Alatau City Hospital, Almaty, Kazakhstan; ^3^Faculty of Medicine, Khoja Akhmet Yassawi International Kazakh-Turkish University, Turkestan, Kazakhstan; ^4^Institute of Social Medicine, Faculty of Medicine, University of Belgrade, Belgrade, Serbia; ^5^Laboratory for Strengthening Capacity and Performance of Health System and Workforce for Health Equity, Faculty of Medicine, University of Belgrade, Belgrade, Serbia; ^6^Vice Rector for Science and Strategic Development, Khoja Akhmet Yassawi International Kazakh-Turkish University, Turkestan, Kazakhstan; ^7^Faculty of Medicine, Institute for Orthopaedics «Banjica», University of Belgrade, Belgrade, Serbia; ^8^Serbian Medical Chamber, Belgrade, Serbia; ^9^Center for Strategic Development, Khoja Akhmet Yassawi International Kazakh-Turkish University, Turkestan, Kazakhstan; ^10^Faculty of Medicine, Khoja Akhmet Yassawi International Kazakh-Turkish University, Turkestan, Kazakhstan; ^11^Faculty of Foreign Languages, Karaganda Buketov University, Karaganda, Kazakhstan; ^12^School of Sciences and Humanities, Nazarbayev University, Astana, Kazakhstan; ^13^Telemedicine Center, Central City Clinical Hospital, Almaty, Kazakhstan; ^14^Health Research Institute, Al-Farabi Kazakh National University, Almaty, Kazakhstan

**Keywords:** COVID-19, coronavirus infection, children, outpatient, non-pharmaceutical interventions, epidemiology

## Abstract

**Background/objectives:**

During the COVID-19 pandemic, primary health care systems worldwide adapted to manage cases in outpatient settings, including those involving children. The aim of this study was to investigate the epidemiological characteristics of 27,205 outpatient COVID-19 cases among children (0–17 years) in Almaty, Kazakhstan, from 1 January 2021 to 31 December 2022, compared with major epidemiological events and public health measures.

**Methods:**

A cross-sectional analysis was conducted to assess the likelihood of hospitalization regarding demographic characteristics, concomitant diseases, the severity of COVID-19 course, as well as the dynamic of cases.

**Results:**

The majority of children (99.3%) were asymptomatic or mild. Children in the younger age group (0–4) had a higher risk of severe course and hospitalization compared with adolescents aged 15–17 years. Sex and chronic diseases (diabetes mellitus, obesity and chronic obstructive pulmonary disease) did not demonstrate statistical significance. The longest spike in outpatient COVID-19 cases in children coincided with the circulation of Delta and Eta strains, the highest with Omicron.

**Conclusion:**

Among outpatient COVID-19 cases in children, the likelihood of severe forms and hospitalization is higher if the child is under 5 years of age.

## 1 Introduction

The highest number of COVID-19 cases was registered in the age group of 10–14 years (30.4%) overwhelmed the health system, resulting in decrease outpatient services and a shortage of hospital beds (1, 2). In response, outpatient follow-up services for stable patients, including children, have been organized in many countries (3–6). The majority of children experienced milder symptoms than adults and had low rates of hospitalization, making home monitoring a rational, feasible and safe option for children (6, 7). While the mortality rate of children due to COVID-19 has generally been lower than that of adults, it remains a concern for healthcare professionals and public health officials. They share the responsibility of protecting children with coronavirus disease (COVID-19), especially those with pre-existing health conditions, from facing a heightened risk of severe outcomes, such as hospitalizations or death (8). These concerns highlight the importance of ongoing research initiatives that target the pediatric population and ensure effective outpatient healthcare delivery.

International evidence shows that in high-income countries (e.g., USA, UK, Canada) with significantly higher levels of investment in children’s health care, there has been greater availability of testing, vaccination, which in turn has controlled severe cases and mortality rates (9). Innovative forms of pediatric outpatient follow-up have also been successfully implemented in these countries. In the USA, a specialized Care and Respiratory Observation Clinic (CROC) with active monitoring at Stanford University; in South Korea, Pediatric COVID-19 Module Clinics (PMC) at a university hospital; in Greece and Singapore, telemedicine systems with routing algorithms and Home Recovery Programmer (HRP) have been introduced (10–12).

Kazakhstan, despite its status as an upper-middle-income country, actually faces similar constraints as other Central Asian states (e.g., Kyrgyzstan and Uzbekistan), where spending on health care does not exceed 2.5–3 per cent of GDP (Gross Domestic Product) (13, 14). This affected the organization of outpatient care, vaccination coverage, and the availability of medical technology. Nevertheless, these countries have made efforts to adapt pediatric care models based on international experience. For example, in Kyrgyzstan, temporary outpatient clinics were deployed at the height of the pandemic (15). In Kazakhstan, the main response has been the establishment of mobile teams at primary healthcare organizations. More than 3,000 teams provided home-based outpatient care for patients with mild and moderate forms of COVID-19, including children (16). National infection control measures in the country included quarantine restrictions, «Ashyq» (meaning “Open” in Kazakh) digital monitoring system, quarantine restrictions, mass vaccination (17–19). The epidemiological situation in the country varied according to the circulation of SARS-CoV-2 virus strains: in 2021, Alpha and Delta variants dominated, causing a high burden on the health system (19). By 2022, the Omicron variant, with continued morbidity but with gradual lifting of restrictions (20, 21). Although international studies on the clinical features and outcomes of COVID-19 in children are available, data from Central Asia remain limited. Notably, there have been no large-scale analyses of outpatient cases in children, despite the fact that most of these patients are seen in primary settings. To assess the changes in COVID-19 case reporting among children, we analyzed 2 years: 1 year under enhanced quarantine measures (2021) and the other with their gradual removal (2022).

## 2 Materials and methods

### 2.1 Study design and subjects

The present study is a retrospective descriptive analysis including two interrelated areas: analysis of the dynamics of COVID-19 cases among children (0–17 years old) under outpatient care in Almaty city in the period from 1 January 2021 to 31 December 2022, covering 27,205 outpatient cases, as well as assessment of demographic and clinical characteristics of outpatients depending on age, sex and concomitant disease. Sex and severity of COVID-19 were considered as the main factors for comparison in this study based on existing literature indicating possible differences in susceptibility, immune response and clinical outcomes in children (22). Previous studies have shown that male children may have a slightly higher risk of infection or severe outcomes, and comorbidities may influence disease progression (22–24).

Almaty is the largest city and economic center of Kazakhstan, with a population of 1,949,726 people (25). In 2021, the proportion of children (aged 0–17 years) in Almaty was 26.7 percent (527,739) and 8.4 percent of the total child population of Kazakhstan.

### 2.2 Case identification

The case of COVID-19 in children was established in accordance with the clinical protocols for the diagnosis and treatment of «COVID-19 Coronavirus infection», which strictly followed the interim recommendations of the World Health Organization (WHO). The diagnosis of COVID-19 is based on a real-time polymerase chain reaction (RT-PCR) – «COVID-19, the virus has been identified» (ICD code 10 – U07.1) and exclusion of alternative diagnoses on the basis of differential diagnosis (26, 27). An outpatient RT-PCR test for coronavirus was performed by taking a swab from the mucous membrane of the mouth and nasopharynx (26, 27). Free RT-PCR testing was available to all symptomatic patients and individuals in contact with confirmed COVID-19 cases. Testing was also available at private laboratories on a fee-for-service basis.

### 2.3 Study variables

The study population included children aged from birth up to their 18th birthday (0–17 years). Individuals who had reached 18 were categorized as adults.

All primary health care organizations in Almaty, including public and private polyclinics (60 institutions in total), transmitted data on COVID-19 cases to the database of the Almaty Telemedicine Centre (TMC), which coordinated the monitoring and control of morbidity, and all outpatient cases were registered in the unified electronic information system of the TMC. TMC provided 24/7 remote monitoring of COVID-19 patients, integrating data from healthcare organizations and promptly sending information to doctors for decision-making. Its functions included keeping records of patient calls, organizing remote consultations, and coordinating the work of mobile teams providing medical care at home (28).

For this study, data from the TMC’s electronic database were obtained from children aged 0 to 17 years, inclusive, who were diagnosed with COVID-19 and observed on an outpatient basis in Almaty from January 01, 2021, to December 31, 2022. With respect to case selection, the electronic TMC database incorporates built-in validation features, which ensure that no record can be saved without essential information, including sex, date of birth (which is automatically converted into age in years), and PCR results indicating whether the outcome is positive or negative. The criteria for exclusion encompassed individuals aged 18 years and older, as well as duplicate records. Consequently, the final dataset comprises a total of 27,205 observations.

All registered cases of COVID-19 coronavirus infection among children receiving outpatient care from mobile team members in 2021–2022 were recorded in the register of TMCs in Almaty city by the staff of polyclinics. The duration of outpatient follow-up for children with coronavirus infection was established according to the recommendations of the clinical protocol, ranging from 10 to 14 days (26, 27).

The dataset included:

- Each case was assigned a unique identifier (ID).
- Age was grouped into four categories: 0–4, 5–9, 10–14, and 15–17.
- Sex categories included boy and girl (assigned at birth).
- ICD X case codes were U07.1 or U07.2.
- Disease severity was classified as asymptomatic, mild, moderate, and severe. Disease severity was determined based on the WHO scale adapted according to the Kazakhstan clinical protocol for diagnosis and treatment (27). The classification of COVID-19 severity relied on clinical presentation, radiographic findings, respiratory parameters, and the presence of concomitant diseases:

◦ Asymptomatic: positive RT-PCR test for SARS-CoV-2 in the absence of any clinical symptoms;
◦ Mild disease: positive RT-PCR test for SARS-CoV-2 in symptomatic patients without evidence of viral pneumonia or hypoxia; SpO₂ is ≥95% on room air; the presence of concomitant diseases is variable (without signs of decompensation);
◦ Moderate disease: positive/negative RT-PCR test; symptomatic patients present with clinical signs of pneumonia (chest X-ray shows hazy darkening, often of rounded morphology, with peripheral and lower distribution in the lungs, and CT scans typically reveal lesions covering up to 50% of the lung area); SpO₂ ≥ 90–95% on room air; concomitant diseases are typically present, without signs of decompensation.
◦ Severe disease: positive or negative RT-PCR test; symptomatic patients with bilateral viral pneumonia, with lung involvement exceeding 50% on CT (computed tomography) imaging; respiratory rate of 30 breaths per minute or greater; severe respiratory distress; or SpO2 less than 90% on room air; concomitant diseases showing evidence of decompensation.
◦ Critical disease: symptomatic patients with acute respiratory distress syndrome, and/or sepsis, and/or septic shock, and/or acute thrombosis, and/or multiple organ dysfunction. Outpatients with critical forms of the disease were excluded from the study, as they were immediately hospitalized.

- Date and RT-PCR result,
- Concomitant diseases (obesity, chronic obstructive pulmonary disease (COPD), diabetes mellitus) based on data from the Electronic Dispensary Patients Register (EDPR) of the Ministry of Health. Diagnoses were recorded in the study database as binary variables (yes/no), with a “yes” response entered only when the diagnosis was confirmed in the EDPR, ensuring consistency and eliminating the risk of subjective assessment by primary care staff.
- Presence of verified pneumonia (chest X-ray), where it is not indicated whether this was the conclusion of a single or a double radiologist assessment, or computed tomography (CT).
- Date and outcome of follow-up [expiry of outpatient follow-up (according to clinical protocol), hospitalization in an infectious disease hospital, hospitalization in other hospitals, patient death (cause unknown), patient moving to another district].

Study approval was obtained centrally from the TMC. The information was anonymized and analyzed in aggregate without patient identification, so informed consent was not required.

For statistical analyses of COVID-19 severity in outpatient children, all clinical cases were grouped into two broad categories: asymptomatic and mild forms in one group and moderate-to-severe and severe forms in the other. A separate analysis of moderate and severe cases was not possible due to the very small number of severe cases (*n* = 1), which would have led to unstable estimates. Additionally, from an outpatient management perspective, both moderate and severe cases are clinically significant because they require closer monitoring compared to asymptomatic and mild forms.

### 2.4 Statistical analysis

The data were analyzed using both descriptive (counts, %) and analytical statistics (Pearson Chi-square test, and multivariate logistic regression). Categorical variables were presented as percentage frequencies, and the Pearson Chi-square test was used to assess differences between groups. In addition, the relationship between sex and age in reported COVID-19 cases was described.

Multivariate logistic regression was applied to identify associations between concomitant disease and COVID-19 severity and hospitalization in infectious disease hospitals. The results were expressed as odds ratios (OR) with 95% confidence intervals (CI). Statistical significance was set at *p*-values 0.05. Statistical analysis was conducted with IBM SPSS Statistics 20 software (SPSS 20, Semey, Kazakhstan). Graphical time series analysis was used to analyze the dynamics of COVID-19 outpatient case registrations among children and adolescents (aged 0–17 years) in Almaty, Kazakhstan, from January 2021 to December 2022, to visualize the dynamics of cases based on weekly data and compare them with key epidemiological and non-pharmaceutical interventions (Table 1).

**Table 1:** Events of COVID-19 in Kazakhstan (2021–2022): chronicle and dates.

Event	Date	Description
2020 year		
The 2nd wave of COVID-19	October 2020 to January 2021	The second spike in the incidence of COVID-19, which peaked in December 2020 and January 2021
2021 year		
Vaccination	February 1, 2021	The beginning of mass vaccination against COVID-19 (for adults) Several types of vaccines have been registered: 1. Vector: Sputnik-V – is an adenovirus vector–based vaccine developed in Russia 2. Inactivated vaccines: QazVac (QazCovid-in) – produced by the Research institute for biological safety problems of the Republic of Kazakhstan. HayatVax (BBIBP-CorV, G42 Healthcare, United Arab Emirates) CoronaVac (Sinovac Biotech, China)
The 3rd wave of COVID-19	March to May 2021	A significant spike in morbidity, which lasted until May 31, 2021. This increase in the incidence was due to the widespread spread of British and South African strains of the virus and is associated with the celebration of a public holiday in March.
Digital control via the «Ashyq» app (the meaning of “Open” in Kazakh)	April 26, 2021	The «Ashyq» application has been implemented, a digital control system for the isolation and movement of citizens that uses an person’s individual identification number and QR codes to determine the risk level of COVID-19 infection (red, yellow, blue, green), these colors indicate the severity of the virus infection
The 4th wave of COVID-19	June to October 2021	The surge is associated with the spread of the Delta strain (Indian) in June, the Eta strain (Nigerian) SARS-CoV-2 in August, the maximum increase was noted in August
Measures in education in large cities	September 01, 2021	Offline education resumed throughout the Republic of Kazakhstan, except for Almaty, where a differentiated approach to quarantine was introduced in organizations of secondary, technical and higher education
Vaccination/revaccination	September 01, 2021	57% of adults in Almaty received fully vaccinated (received 2 doses) of one of the four vaccines (Sputnik V, QazVac, Hayat-Vax, CoronaVac), which together reduced the risk of SARS-CoV-2 infection by 76% and prevented more than 100,000 cases
	November 2021	Vaccination of adolescents (12–17 years old) and pregnant women has begun. RNA vaccine: Comirnaty (Pfizer-BioNTech)
	November 21, 2021	The start of adult revaccination
	end of December 2021	According to Our World in Data, by the end of December 2021, 42.5% of Kazakhstan’s population was fully vaccinated (two doses)
2022 year		
January 2022 protests	January 2–19, 2022	On January 2, 2022, due to rising gas prices, mass protests broke out across the country, especially in Almaty, where they escalated into riots and the seizure of government buildings. On January 5, a state of emergency was declared, the government resigned, and on January 6, the forces of the Collective Security Treaty Organization arrived in the country. By January 7, order was restored, and the state of emergency was lifted on January 19
The 5th wave of COVID-19	From January 6, 2022	5th wave of COVID-19 (Omicron strain) has become the largest in terms of the number of new COVID-19 cases, exceeding 15,000 per day
Easing restrictions	From January 27, 2022	Quarantine measures have been relaxed in Ashyq in accordance with its status. With the green status (vaccination/negative PCR) for staff and visitors, facilities can operate without time limits. Restrictions on transport, hotels, train stations, banks, and markets have also been differentially lifted
	February 2022	Restrictions on the offline operation of organizations and offices have been lifted, and it is allowed to hold any events (conferences, forums) at facilities using the Ashyq system to ensure security
	March 2022	It is allowed to hold public events (weddings, concerts, sports events) at Ashyq facilities; From March 20 to March 23, the celebration of the National Holiday Nauryz; March 25, 2022: Resolution №16 abolished the mandatory wearing of masks, except for medical organizations. Masks are recommended in crowded areas and transport
The 6th wave of COVID-19	July 2022	An increase in the incidence of COVID-19 has been recorded in Kazakhstan. As a result, restrictive measures have been temporarily restored in Almaty since July 4, including the mandatory wearing of masks in closed public places (markets, banks, cinemas, public transport, etc.)
Vaccination rate	by December 2022	As of July 27, 2022, the proportion of the fully vaccinated (2 doses) population of the country was 52.46%, and by December 6, 2022, this figure was 52.98%

The data was collected by calendar week and descriptive statistics were used for analysis: the average, minimum (Min), maximum (Max) and standard deviation (SD) for each major epidemic wave. The waves were determined based on a sharp increase in the number of cases per week and compared with the periods of spread of known variants (Alpha (British) and Beta (South African), Delta, Eta (Nigerian), Omicron) during the analyzed period. The comparative analysis was carried out by calculating the relative increase in the average number of weekly cases during each wave compared to the baseline before the British strain was identified in early 2021 (February), which was used as a starting point.

## 3 Results

**Figure 1:**
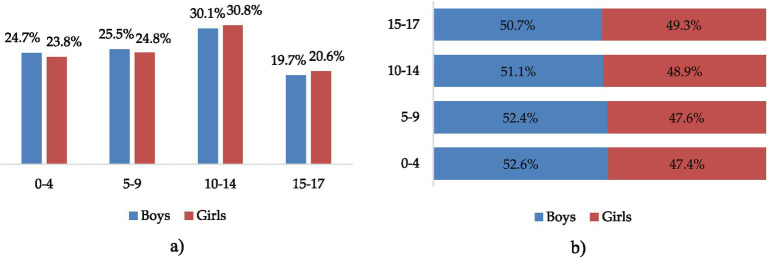
Distribution of COVID-19 outpatient cases by sex and age groups, 2021 to 2022 in Almaty, %. **(a)** By sex and age group. **(b)** Sex distribution within age groups.

Between 2021 and 2022, 27,205 children aged 0 to 17 years with COVID-19 were under outpatient follow-up in Almaty city. Among them, 14,064 (51.7%) were boys and 13,141 (48.3%) were girls. The highest number of COVID-19 cases was registered in the age group of 10–14 years (30.4%) (Figure 1A). In each age group, boys accounted for slightly more than half of the reported cases (Figure 1B).

However, the statistical analysis showed no significant association between sex and the distribution of outpatient COVID-19 cases across age groups (χ^2^ = 6.541, *p* > 0.05), with a relatively balanced distribution between boys and girls in all categories (0–4, 5–9, and 10–14 years), and no significant sex differences in the frequency of comorbidities (*p* > 0.05) (Table 2).

**Table 2:** Characteristics of outpatient COVID-19 cases among children in Almaty by sex, age and comorbidities (2021–2022), (*n* = 27,205, 100%).

Outpatient characteristics		COVID-19 outpatient cases			Test of difference	
		Total (*n* = 27,205)	Boys (*n* = 14,064)	Girls (*n* = 13,141)	χ^2^	*p*-value
Age group (years)	0–4	6,606 (24.3%)	3,473 (24.7%)	3,133 (23.8%)	6.541	0.089*
	5–9	6,840 (25.1%)	3,581 (25.5%)	3,259 (24.8%)		
	10–14	8,271 (30.4%)	4,228 (30.1%)	4,043 (30.8%)		
	15–17	5,488 (20.2%)	2,782 (19.8%)	2,706 (20.6%)		
Concomitant disease (diagnosed)						
Diabetes mellitus ICD-X: E10-E10.8	30 (0.1%)		14 (0.1%)	16 (0.1%)	0.304	0.590*
Obesity ICD-X: E66-E67	28 (0.1%)		16 (0.1%)	12 (0.1%)	0.333	0.557*
COPD ICD-X: J44	15 (0.1%)		9 (0.1%)	6 (0.05%)	0.414	0.610*

*Chi-squared test.

Out of the total reported outpatient pediatric COVID-19 cases, 99.3% (*n* = 27,023) experienced mild or asymptomatic disease, while only 0.7% (*n* = 182) developed a moderate to severe form of the infection, including only one case of a severe form. When examining the distribution by age groups, it was observed that younger children (aged 0–4 years) were more frequently represented in the category with a severe course of COVID-19, accounting for 39.0% of severe cases (*p* < 0.001). Moreover, no significant differences were found between different sex groups regarding the severity of COVID-19 (*p* = 1.0), suggesting that our study population had similar numbers of boys and girls across both severity levels. Additionally, there were no statistically significant differences in the severity groups regarding the presence of comorbidities such as diabetes mellitus, obesity, and COPD (*p* > 0.05 for each). These conditions were exclusively found among children with asymptomatic and mild cases of COVID-19 (Table 3).

**Table 3:** Distribution of pediatric outpatients, by age, sex, and concomitant disease according to the severity of COVID-19, Almaty (2021–2022).

Outpatient characteristics (*n* = 27,205, 100%)		Severity course COVID-19					Test for the difference	
		Total (*n* = 27,205)	Asympt + Mild (*n* = 27,023)		Moderate + Severe (*n* = 182)		χ2	*p*-value
		*n* (100%)	*n*	%	*n*	%		
Age group (years)	0–4	6,606 (24.3)	6,535	24,2	71	39,0	23.823	<0.001
	5–9	6,840 (25.1)	6,795	25,1	45	24,7		
	10–14	8,271 (30.4)	8,232	30,5	39	21,4		
	15–17	5,488 (20.2)	5,461	20,2	27	14,8		
Sex	Boys	14,064 (51.7)	13,970	51.7	94	51.6	0.0	1.0
	Girls	13,141 (48.3)	13,053	48.3	88	48.4		
Diabetes mellitus ICD-X: E10-E10.8		30 (0.1)	30,0	0.1	0,0	0,0	0.202	1.0
Obesity ICD-X: E66-E67		28 (0.1)	28,0	0.1	0,0	0,0	0.189	1.0
COPD ICD-X: J44		15 (0.1)	15,0	0.1	0,0	0,0	0.101	1.0

During the study period, 94.8% (*n* = 25,782) of children with COVID-19 under home observation were discharged after the observation period ended.

### 3.1 Multivariate regression analysis

Among outpatient children with COVID-19 in Almaty during 2021–2022, only the age group of 0–4 years showed a statistically significant association with an increased risk of developing moderate or severe disease, with a 2.2-fold increase compared to adolescents aged 15–17 years (OR = 2.197; 95% CI: 1.409–3.428; *p* = 0.001). Other age groups (5–9 and 10–14 years), as well as male sex (OR = 1.011; 95% CI: 0.755–1.354; *p* = 0.940), were not significantly associated with increased disease severity. Furthermore, the presence of concomitant diseases such as obesity, diabetes mellitus, and COPD were not found to be significantly associated with disease severity (Table 4). This lack of association may be attributed to the low number of reported outpatient cases with these comorbidities in Almaty, but it might be more prevalent in hospitals.

**Table 4:** Multifactorial analysis of potential predictors of moderate and severe COVID-19 among outpatient children in Almaty (2021–2022), *n* = 182.

Influence variables		Outcome variables	
		Moderate and Severe Course of COVID-19	
		Odds Ratio (95% Confidence Interval)	*p*-value
Age intervals	0–4	2.197 (1.409–3.428)	0.001
	5–9	1.339 (0.830–2.161)	0.231
	10–14	0.958 (0.586–1.567)	0.865
	15–17	Reference category	
Sex	Boys	1.011 (0.755–1.354)	0.940
Concomitant disease (diagnosed)	Diabetes mellitus ICD-X: E10-E10.8	–	0.998
	Obesity ICD-X: E66-E67	–	0.998
	COPD ICD-X: J44	–	0.999

Among outpatient children with COVID-19 in Almaty during 2021–2022, multivariate logistic regression analysis revealed that children aged 0–4 years had a statistically significant 2.4-fold increase in the odds of being hospitalized in an infectious disease hospital compared to adolescents aged 15–17 years (OR = 2.370; 95% CI: 1.943–2.891; *p* < 0.001). The odds of hospitalization were approximately 2.7 times lower among children with asymptomatic or mild COVID-19 than among those with moderate or severe disease (OR = 0.370; 95% CI: 0.223–0.615; p < 0.001). Sex (OR = 1.098; 95% CI: 0.960–1.256; *p* = 0.171) and the assessed concomitant disease, including obesity, diabetes mellitus, and COPD were not significantly associated with increased hospitalization risk (Table 5).

**Table 5:** Multifactorial analysis of predictors of hospitalization in an infectious disease hospital among outpatient children with COVID-19 in Almaty (2021–2022), *n* = 888.

Influence variables		Outcome variables	
		Hospitalization in an infectious diseases hospital	
		Odds ratio (95% confidence interval)	*p*-value
Age intervals	0–4	2.370 (1.943–2.891)	<0.001
	5–9	1.023 (0.815–1.283)	0.845
	10–14	0.943 (0.756–1.177)	0.606
	15–17	Reference category	
Sex	Boys	1.098 (0.960–1.256)	0.171
Concomitant disease (diagnosed)	Diabetes mellitus ICD-X: E10-E10.8	–	0.998
	Obesity ICD-X: E66-E67	–	0.998
	COPD ICD-X: J44	–	0.999
Severity course COVID-19	Asympt + mild	0.370 (0.223–0.615)	<0.001
	Moderate + Severe	Reference category	

### 3.2 Analysis of the dynamics of COVID-19 outpatient cases

**Figure 2:**
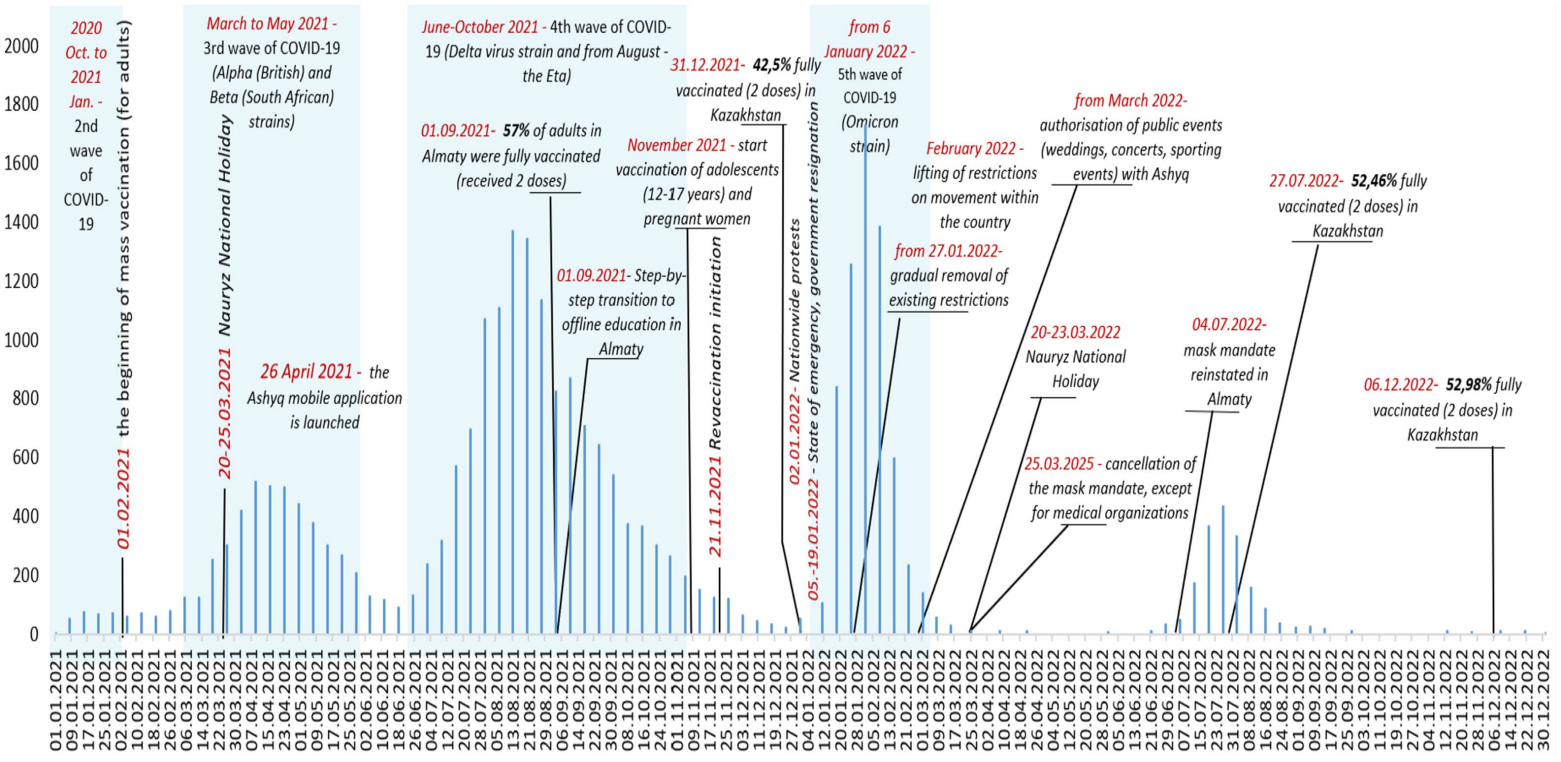
Dynamics of outpatient COVID-19 cases in children, key events and interventions, Almaty, 2021–2022.

The analysis of the dynamics of outpatient cases of COVID-19 among children in Almaty from 01 January 2021 to 31 December 2022 demonstrates a wave pattern of coronavirus infection, featuring pronounced peaks and periods of decline in the number of cases (Figure 2).

In 2021, the number of cases was 2.6 times higher than in 2022, comprising 69.9% of all cases compared to 30.1%. During the study period, there were four spikes in outpatient COVID-19 cases among children and adolescents (Figure 2). At the beginning of 2021, the average number of reported coronavirus infections among children at the outpatient level was 72.6 per week. The first spike occurred in the first quarter of 2021 with a peak in April; during this period, the average weekly caseload for outpatient services increased 4.8 times compared to the beginning of the year to 350.3 cases (Figure 2).

The longest sustained surge of SARS-CoV-2 infection among outpatients in Almaty, Kazakhstan, was recorded during the fourth wave associated with the Delta and Eta strains of the virus in the summer of 2021. This surge lasted for approximately 4 months, from mid-June to the end of October 2021 (Figure 2). The peak in the registration of outpatient pediatric cases of coronavirus infection occurred in August 2021, when the weekly number of cases reached 1,372. During this period, the average weekly caseload for the city’s outpatient service was 654.9 COVID-19 cases among children and adolescents, which was 1.9 times the level of the previous wave (Table 6). Overall, the most burdensome period of the pandemic for outpatient services in Almaty was the period caused by the Delta Wave, which was characterized by both consistently high weekly COVID-19 case numbers (ranging from 500 to 1,372) and its prolonged duration. The surge coincided with the beginning of the academic year (September 1), when 57% of the adult population in Almaty had been fully vaccinated and a step-by-step return to in-person education had begun (Figure 2).

**Table 6:** Comparison of the average weekly outpatient COVID-19 cases among children in different periods of strain circulation, Almaty, Kazakhstan, 2021–2022.

Wave	Period	Mean weekly cases	Min*	Max**	SD***	Fold increase compared to the initial level
Pre-Alpha and Beta	February 2021	72.6	47	120	19.71	Reference
Alpha (British) and Beta (South African)	March to May 2021	350.3	112	546	132.80	4.8×
Delta (Indian) and Eta (Nigerian)	June to October 2021	654.9	129	1,404	390.07	9.0×
Omicron strain	January 2022	758.8	7	1793	663.18	10.5×

*Minimum, **Maximum, ***standard deviation.

The highest spike in COVID-19 case reporting among children at the outpatient level was due to the fifth wave of the pandemic associated with the spread of the Omicron strain in January 2022. This surge was particularly dramatic following nationwide protests and the subsequent declaration of a national state of emergency (2–19 January 2022), coinciding with the start of the phase-out of restrictive measures from 27 January 2022 (Figure 2). For the week beginning 25 January 2022, there were 1,731 cases and an average of 758.8 cases per week, 1.2 times the delta wave rate, and 10.5 times higher than in early 2021 (Table 6). Although the surge itself was short-lived, its intensity was significant (Figure 2).

After the cancellation of the mandatory face mask requirement for COVID in March 2022, there was only one spike in cases in July, the smallest and shortest of all four, likely due to a combination of all coronavirus variants.

After each upsurge, the number of cases decreased successively, and since September 2022, the registration of outpatient pediatric COVID-19 cases in Almaty has remained at a minimum level (Figure 2).

## 4 Discussion

In this study, we investigated the epidemiological characteristics of COVID-19 among children aged 0–17 years in Almaty who were registered as COVID-19 cases in the outpatient care follow-up services during the consecutive pandemic years of 2021 and 2022. We also analyzed whether the frequency and severity of cases and hospitalizations vary significantly in relation to the type of virus, age interval, sex, or comorbidity among children, considering the impact of preventive measures imposed to safeguard children’s health during the pandemic in Kazakhstan. The likelihood of hospitalization was higher for children under 5 years old and those with severe COVID-19. In Almaty, there was the possibility that one child aged 0–17 years would be registered with severe COVID-19 in 143 cases. The odds of severe forms and hospitalization would be twofold higher if a child is under 5 years of age.

Analysis of sex distribution showed a slight predominance of boys. Early studies revealed a higher incidence of COVID-19 among boys in the general population of children (0–17 years) (29). However, in the present study, no statistically significant differences were found for sex and severity of the course of coronavirus infection, nor for hospitalization, in the age groups of outpatient children with COVID-19 (0–4, 5–9, 10–14, 15–17 years).

In our study in Almaty, we observed that children with mild or asymptomatic COVID-19 cases were predominantly seen at outpatient facilities. This aligns with the trend of children experiencing milder symptoms compared to adults (30, 31). However, the risk of severe coronavirus infection in childhood might increase when other conditions, such as obesity, COPD, or diabetes mellitus, are present (23, 24). In our study sample of outpatient cases of coronavirus infection among children, this relationship was not observed, probably due to the low prevalence of chronic diseases. The newly developed Pediatric Comorbidity Index indicates that there is a low prevalence of comorbidities in hospitalized children, with the exceptions of injuries, pain, asthma, and obesity (32). This finding may help explain the low occurrence of concurrent diseases observed in our study group. Other factors that could contribute to this result include underreporting and the decision to focus solely on outpatient cases, excluding those who were already hospitalized. Future studies similar to this may benefit from implementing a pediatric comorbidity score to minimize confounding bias and more accurately evaluate risks.

Children aged 0–4 years were significantly more likely to experience moderate and severe COVID-19 cases during home observation in Almaty, Kazakhstan, with a hospitalization risk 2.4 times higher for this age group. Several studies have observed similar age-specific patterns in the pediatric population. Children under 3 years are more vulnerable to severe forms of COVID-19 compared to older children aged 12–17 years (33). Children aged 4 to 6 years, especially those under 1 year, are at a high risk of severe COVID-19. Additionally, hospitalization rates are higher for those under 2 years of age compared to older children and adolescents (34–36). Although age is a significant risk factor for developing severe COVID-19 in the general population due to immune aging and age-related diseases (37), the relationship between age and disease severity in children seems to be the opposite. This difference highlights the unique aspects of COVID-19 infection in children. Consequently, this difference is crucial for informing and shaping public health policies aimed at mitigating the effects of COVID-19 on children and ensuring their well-being during the pandemic.

A comprehensive systematic review indicated that a notable percentage of pediatric cases necessitate hospital admission for moderate to severe COVID-19 (23). A study in the Republic of Moldova reported an increase in hospitalization of COVID-19 moderate to severe pediatric cases up to 25.7% during waves of the Delta and Omicron variants (January 2021 to February 2022) (38). In our study, in the same period, 5.2% of all children treated on an outpatient basis were admitted to infectious disease hospitals. The likelihood of hospitalization was higher among those with severe forms of the illness. The easing of quarantine measures and an increase in social interactions helped spread the British and South African variants of the virus, alpha, Delta strain, and the Eta, resulting in significant pressure on healthcare services in the spring and summer of 2021 and the winter of 2022 (19, 39). Most of the children involved in our study experienced positive treatment outcomes, which indicates the effectiveness of outpatient care.

In the USA, the alpha wave peaked in late 2020-early 2021 and was accompanied by a significant increase in mortality rates (40). However, unlike in the US, where vaccination began in January 2021, in Kazakhstan, the campaign started later, in February 2021, and primarily targeted at-risk groups. At the time of the third wave, children were not yet in the vaccination groups, which increased their susceptibility to the virus (19). The introduction of the digital system “Ashyq,” linked to the individual identification number of a citizen, using QR code technology, has strengthened infection control (17, 18).

However, the global dominance of the Delta strain and the Eta (Nigerian) strain of SARS-CoV-2 in the summer of 2021 has led to a new outbreak in both high healthcare spending countries (USA, France, UK), and resource-limited countries, such as Kazakhstan and neighboring Uzbekistan (20, 41, 42). The Delta SARS-CoV-2 variant was highly transmissible and virulent, causing a more severe course of the disease, increased risk of hospitalization, and need for intensive care, which significantly increased the burden on the health care system (43, 44). The primary health care service also felt the strain, with registration of outpatient COVID-19 cases among Almaty children rising sharply from July, peaking in August. Despite the increase in cases, high-income countries achieved lower mortality rates, attributed to high vaccination rates (up to 98%) (45). Whereas, in Uzbekistan, where health spending is comparable to Kazakhstan, the 2021 summer surge accounted for about 60% of all deaths during the pandemic period (42). Also, in Kazakhstan, the main burden of years of life lost (YLL) in 2021 fell on the summer and autumn months (46).

However, after peaking in August, the number of reported outpatients COVID-19 cases among children in Almaty, Kazakhstan, gradually declined, reaching a low in December. Although children were not eligible for vaccination, a positive epidemiological effect may have been achieved by increasing collective immunity: by September 2021, more than half (57%) of the city’s adult population had received a second dose of vaccine, and the four vaccines administered showed a combined efficacy in reducing the risk of infection (17). Nevertheless, the partial return to full-time schooling in September may have kept the virus circulating in the pediatric population (47). Subsequently, in November 2021, Kazakhstan started vaccination of adolescents aged 12 years and older, pregnant women, and started a revaccination campaign for adults (48). According to Our World in Data, by the end of December 2021, 42.5 per cent of the country’s population had received a full course of vaccination (two doses) (49).

In late 2021 and early 2022, the Omicron strain quickly displaced Delta and became dominant in many countries with high GDP for healthcare, such as the UK and the US, due to its high transmissibility and partial immune escape (50–52). Despite this, it generally caused mild illness, particularly in highly vaccinated populations. In response, the UK reintroduced masking, isolation, and expanded booster campaigns (53). The situation in Almaty, Kazakhstan, largely repeated the global trends related to the spread of the Omicron variant. In January 2022, similar to high-income countries, including the UK, there was a sharp increase in outpatient cases of COVID-19 in children, likely due to the sub-variant’s high contagiousness, short incubation period, and mild course (20, 21, 54). However, unlike in the UK, where a second wave caused by BA.2 with a peak in the older adults was reported in March, Almaty, Kazakhstan, has seen a rapid decline in COVID-19 cases in children at outpatient level since February, despite political instability (mass protests and state of emergency from 5 to 19 January), subsequent relaxation of restrictions and public events (e.g., Nauryz in March) (55–57).

The next increase in outpatient pediatric cases in Almaty, Kazakhstan, in our study was in July 2022, and in response, the city authorities temporarily reintroduced restrictive measures, including mandatory indoor masking, in response to our findings (58). Increased social contacts during summer holidays and vacations, foreign travel, and decreased adherence to preventive measures in the context of canceled restrictions, as well as the possible emergence of new Omicron sub-variants, could have played a significant role in the spread of infection. However, it is difficult to accurately assess the contribution of each of these due to changing testing levels, population behavior, administrative decisions, and possible underestimation of official data (20). Thus, in the USA, the fifth epidemiological wave recorded between 7 March and 18 July 2022 was due to the circulation of Omicron BA.2 and BA.5 sub-variants (59).

Thus, the observed fluctuations in morbidity among children in Almaty in 2022 reflect the influence of both epidemiological factors and social conditions. Against this background, in this study, we first examined cases of coronavirus infection in a pediatric population who were in home isolation under outpatient care supervision for the period from January 2021 to December 31, 2022, in the largest metropolitan area of Kazakhstan, Almaty. From a public health policy perspective, this study highlights the vital role of outpatient services in managing the epidemic among children in Kazakhstan and similar settings. It is essential to continuously strengthen the capacity of outpatient services to be prepared to facilitate vaccination, ensure early disease detection, actively monitor children with mild or asymptomatic cases, manage patients at home, and provide timely hospitalization when necessary. These efforts will help alleviate the burden on inpatient facilities and reduce the amount of time patients spend in hospitals during epidemics. However, it remains crucial to monitor the long-term effects of COVID-19 on children and to develop innovative approaches to pediatric outpatient care.

### 4.1 Limitation

This study focuses on outpatient COVID-19 cases in children and does not include patients treated in hospitals, such as those with critical illness who were immediately hospitalized. This narrows the possibility of analyzing the entire clinical picture of the disease. In addition, children who did not seek medical care or stopped follow-up were not included. The lack of information on those who did not continue treatment limits the evaluation of the effectiveness of preventive and telemedicine interventions. The length of follow-up and possible errors in the classification of disease severity, as well as possible underreporting of concomitant disease, may affect the accuracy of conclusions. In addition, incomplete reporting of morbidity trends is associated with changes in testing levels, population behavior, and administrative decisions, making it difficult to accurately assess the impact of these factors on morbidity. The two-year follow-up period does not fully capture the long-term effects of COVID-19 in children, which requires further research.

## 5 Conclusion

The study showed that in 2021–2022, the majority of COVID-19 cases among children in Almaty were mild or asymptomatic. However, against the background of epidemic waves and the circulation of new strains of the virus, there were periods of increased cases and an increased burden on outpatient care. The greatest risk of severe disease and hospitalization was observed in young children (0–4 years of age). Despite the presence of comorbidities, there was no significant association with the severity of coronavirus infection.

Although vaccination of children in Kazakhstan started later, the formation of collective immunity among adults and restrictive measures apparently played an important role in stabilizing the epidemiological situation in the child population. Comparisons with high-income countries show that, despite more limited resources, Kazakhstan generally followed global epidemiological trends, although differences in vaccination coverage and the resilience of health systems may have influenced the magnitude and impact of individual pandemic waves.

The effectiveness of outpatient care during the pandemic is evident in the fact that a significant number of children were able to receive care at home, which helped reduce the risk of overburdening the healthcare system.

Prospects for further research include more comprehensive studies of outpatient COVID-19 cases in children, including a larger set of clinical characteristics, transmission patterns, and long-term outcomes.

## Data Availability

The raw data supporting the conclusions of this article are available from the corresponding author, upon reasonable request.

## Glossary

COVID-19: Coronavirus Disease 2019
USA: United States of America
UK: United Kingdom
CROC: Care and Respiratory Observation Clinic
PMC: Pediatric Module Clinic
HRP: Home Recovery Programme
GDP: Gross Domestic Product
WHO: World Health Organization
RT-PCR: Real-Time Polymerase Chain Reaction
TMC: Telemedicine Center
ICD X: International Classification of Diseases 10th Revision
SARS-CoV-2: Severe Acute Respiratory Syndrome Coronavirus 2
SpO₂: Saturation Peripheral Oxygen
X-ray: X-radiation
CT: Computed Tomography
COPD: Chronic Obstructive Pulmonary Disease
EDPR: Electronic Dispensary Patients Register
OR: Odds Ratio
CI: Confidence Interval
IBM: International Business Machines Funding
SPSS: Statistical Package for the Social Sciences
Min: Minimum
Max: Maximum
SD: Standard Deviation
QR: Quick Response
Asymp: Asymptomatic
YLL: Years of Life Lost

## References

[1] Dupraz J Le Pogam M-A Peytremann-Bridevaux I. Early impact of the COVID-19 pandemic on in-person outpatient care utilisation: a rapid review. BMJ Open. (2022) 12:e056086. doi: 10.1136/bmjopen-2021-056086, PMID: 35241471 PMC8895419

[2] Glushkova N Semenova Y Sarria-Santamera A. Editorial: public health challenges in post-soviet countries during and beyond COVID-19. Front Public Health. (2023) 11:1290910. doi: 10.3389/fpubh.2023.1290910, PMID: 37886052 PMC10598333

[3] Liapikou A Tzortzaki E Hillas G Markatos M Papanikolaou IC Kostikas K. Outpatient management of COVID-19 disease: a holistic patient-centered proposal based on the Greek experience. J Pers Med. (2021) 11:709. doi: 10.3390/jpm11080709 34442353 PMC8400346

[4] Artandi M Barman L Srinivasan M Thomas S Singh J Asch SM. A Specialized Acute COVID-19 Outpatient Clinic at an Academic Medical Center. Am J Med Qual. (2022) 37:221–6. doi: 10.1097/JMQ.0000000000000006, PMID: 34310381 PMC9052357

[5] Blair PW Brown DM Jang M Antar AAR Keruly JC Bachu VS. The clinical course of COVID-19 in the outpatient setting: a prospective cohort study. Open Forum Infect Dis. (2021) 8:ofab007. doi: 10.1093/ofid/ofab007 PMC7881750 33614816

[6] Yun KW Kim KM Kim YK Kim MS Kwon H Han MS. Limited benefit of facility isolation and the rationale for home Care in Children with mild COVID-19. J Korean Med Sci. (2021) 36:e45. doi: 10.3346/jkms.2021.36.e45, PMID: 33527787 PMC7850862

[7] Vergine G Fantini M Marchetti F Stella M Valletta E Biasucci G. Home Management of Children with COVID-19 in the Emilia-Romagna region, Italy. Front Pediatr. (2020) 8:575290. doi: 10.3389/fped.2020.575290, PMID: 33194906 PMC7644844

[8] Mansourian M Ghandi Y Habibi D Mehrabi S. COVID-19 infection in children: a systematic review and meta-analysis of clinical features and laboratory findings. Arch Pediatr. (2021) 28:242–8. doi: 10.1016/j.arcped.2020.12.008 33483192 PMC7794595

[9] da Fonseca Lima EJ Leite RD. COVID-19 vaccination in children: a public health priority. J Pediatr. (2023) 99:S28–36. doi: 10.1016/j.jped.2022.11.006 PMC9767816 36564007

[10] Tan T Yau JWK Toh MPHS Vasoo S Leo YS. Coronavirus disease and home recovery: a Singapore perspective. Western Pacific Surveillance Response J. (2023) 14:09–15. doi: 10.5365/wpsar.2023.14.5.1003 PMC10632601 37969814

[11] Choe YJ Lee JS Lee Y Park KH Yoo Y Im G-J. Building of pediatric COVID-19 module clinic: a novel operation model in response to COVID-19 pandemic. J Korean Med Sci. (2023) 38:1–7. doi: 10.3346/jkms.2023.38.e96, PMID: 37012684 PMC10070052

[12] Smyrnakis E Symintiridou D Andreou M Dandoulakis M Theodoropoulos E Kokkali S. Primary care professionals’ experiences during the first wave of the COVID-19 pandemic in Greece: a qualitative study. BMC Fam Pract. (2021) 22:174. doi: 10.1186/s12875-021-01522-9 34474684 PMC8412972

[13] Zhao F Che J Jiawei W Liu Z Li J Zhenis A. Research on health expenditure in Kazakhstan. Central Asia Observ. (2024) 1:1–9. doi: 10.62432/CAO.1.24.0210

[14] Zhamantayev O Kayupova G Nukeshtayeva K Yerdessov N Bolatova Z Turmukhambetova A. COVID-19 pandemic impact on the maternal mortality in Kazakhstan and comparison with the countries in Central Asia. Int J Environ Res Public Health. (2023) 20:2184. doi: 10.3390/ijerph20032184, PMID: 36767550 PMC9914964

[15] Moldokmatova A Dooronbekova A Jumalieva C Estebesova A Mukambetov A Ibragimov S. Mathematical modelling projections versus the actual course of the COVID-19 epidemic following the Nationwide lockdown in Kyrgyzstan. J Biotechnol Biomed. (2023) 6:1–26. doi: 10.26502/jbb.2642-9128008136970578

[16] Gazezova S Nabirova D Detmar A Smagul M Kasabekova L Zikriyarova S. Therapies for people hospitalized with COVID-19 and alignment with national clinical guidelines in a large hospital, Almaty, Kazakhstan, 2020–2021. Front Med (Lausanne). (2023) 10:1248959. doi: 10.3389/fmed.2023.1248959, PMID: 37828941 PMC10566366

[17] Nabirova D Horth R Smagul M Nukenova G Yesmagambetova A Singer D. Effectiveness of four vaccines in preventing SARS-CoV-2 infection in Almaty, Kazakhstan in 2021: retrospective population-based cohort study. Front Public Health. (2023) 11:1205159. doi: 10.3389/fpubh.2023.1205159, PMID: 37351091 PMC10282771

[18] Haruna UA Amos OA Gyeltshen D Colet P Almazan J Ahmadi A. Towards a post-COVID world: challenges and progress of recovery in Kazakhstan. Public Health Challenges. (2022) 1:1–7. doi: 10.1002/puh2.17 PMC12039651 40496378

[19] Semenova Y Kalmatayeva Z Oshibayeva A Mamyrbekova S Kudirbekova A Nurbakyt A. Seropositivity of SARS-CoV-2 in the population of Kazakhstan: a Nationwide Laboratory-based surveillance. Int J Environ Res Public Health. (2022) 19:2263. doi: 10.3390/ijerph19042263, PMID: 35206453 PMC8872132

[20] Sarria-Santamera A Abdukadyrov N Haruna UA Glushkova N Semenova Y Salpynov Z. Estimating the real impact of the COVID-19 pandemic in Kazakhstan: factors associated with detection of the “true infections”. Adv Exp Med Biol. (2024) 1457:373–84. doi: 10.1007/978-3-031-61939-7_21 39283438

[21] Cui Q Shi Z Yimamaidi D Hu B Zhang Z Saqib M. Dynamic variations in COVID-19 with the SARS-CoV-2 omicron variant in Kazakhstan and Pakistan. Infect Dis Poverty. (2023) 12:18. doi: 10.1186/s40249-023-01072-5 36918974 PMC10014408

[22] Kozak K Pavlyshyn H Kamyshnyi O Shevchuk O Korda M Vari SG. The relationship between COVID-19 severity in children and Immunoregulatory gene polymorphism. Viruses. (2023) 15:2093. doi: 10.3390/v15102093 37896870 PMC10612096

[23] Choi JH Choi S-H Yun KW. Risk factors for severe COVID-19 in children: a systematic review and Meta-analysis. J Korean Med Sci. (2022) 37:1–14. doi: 10.3346/jkms.2022.37.e35 PMC8822112 35132841

[24] Fotea S Ghiciuc CM Stefanescu G Cianga AL Mihai CM Lupu A. Pediatric COVID-19 and diabetes: an investigation into the intersection of two pandemics. Diagnostics. (2023) 13:2436. doi: 10.3390/diagnostics13142436, PMID: 37510181 PMC10378192

[25] World Bank UNCG. Almaty, Kazakhstan Population Available online at: https://populationstat.com/kazakhstan/almaty (Accessed April 6, 2025).

[26] Ministry of Healthcare of the Republic of Kazakhstan. Clinical guidelines for diagnosis and treatment of Coronovirus disease, COVID-19. (10.2th edition with additions). (2020). Available at: https://www.gov.kz/memleket/entities/dsm/press/article/details/16275?lang=en (Accessed April 06, 2024)

[27] Clinical protocols of the Ministry of Health of the Republic of Kazakhstan - 2020. Clinical protocol for diagnosis and treatment of “COVID-19 coronavirus infection in children”. Nur-Sultan, Kazakhstan: Ministry of Health of the Republic of Kazakhstan. (2020).

[28] Do TD Gui MM Ng KY. Assessing the effects of time-dependent restrictions and control actions to flatten the curve of COVID-19 in Kazakhstan. PeerJ. (2021) 9:e10806. doi: 10.7717/peerj.10806, PMID: 33604187 PMC7866903

[29] Kozhekenova N Moiynbayeva S Jeremic D Dinic M Semenov P Nurgaliyeva Z. The burden of COVID-19 in primary care of Almaty, Kazakhstan, 2021–2022. Sci Rep. (2025) 15:5186. doi: 10.1038/s41598-025-89707-5 39939733 PMC11822126

[30] Han X Li X Xiao Y Yang R Wang Y Wei X. Distinct characteristics of COVID-19 infection in children. Front Pediatr. (2021) 9:619738. doi: 10.3389/fped.2021.619738 33748041 PMC7969512

[31] Bhuiyan MU Stiboy E Hassan MZ Chan M Islam MS Haider N. Epidemiology of COVID-19 infection in young children under five years: a systematic review and meta-analysis. Vaccine. (2021) 39:667–77. doi: 10.1016/j.vaccine.2020.11.078, PMID: 33342635 PMC7833125

[32] Sun JW Bourgeois FT Haneuse S Hernández-Díaz S Landon JE Bateman BT. Development and validation of a pediatric comorbidity index. Am J Epidemiol. (2021) 190:918–27. doi: 10.1093/aje/kwaa244, PMID: 33124649

[33] Liu Y Xu L Piao X Li H Shi L Huang Y. Epidemiological, clinical, and household transmission characteristics of children and adolescents infected with SARS-CoV-2 omicron variant in Shanghai, China: a retrospective, multicenter observational study. Int J Infect Dis. (2023) 129:1–9. doi: 10.1016/j.ijid.2023.01.030 36724865 PMC9884399

[34] Graff K Smith C Silveira L Jung S Curran-Hays S Jarjour J. Risk factors for severe COVID-19 in children. Pediatr Infect Dis J. (2021) 40:e137–45. doi: 10.1097/INF.0000000000003043 33538539

[35] Bellino S Punzo O Rota MC Del Manso M Urdiales AM Andrianou X. COVID-19 disease severity risk factors for pediatric patients in Italy. Pediatrics. (2020) 146:1–10. doi: 10.1542/peds.2020-009399, PMID: 32665373

[36] Marks KJ Whitaker M Anglin O Milucky J Patel K Pham H. Hospitalizations of children and adolescents with laboratory-confirmed COVID-19 — COVID-NET, 14 states, July 2021–January 2022. MMWR Morb Mortal Wkly Rep. (2022) 71:271–8. doi: 10.15585/mmwr.mm7107e4, PMID: 35176003 PMC8853476

[37] Fonseca DLM Filgueiras IS Marques AHC Vojdani E Halpert G Ostrinski Y. Severe COVID-19 patients exhibit elevated levels of autoantibodies targeting cardiolipin and platelet glycoprotein with age: a systems biology approach. NPJ Aging. (2023) 9:21. doi: 10.1038/s41514-023-00118-0 37620330 PMC10449916

[38] Selevestru R Rotaru-Cojocari D Goloborodico A Bozadji V Negru I Palii I. Evolution of the frequency cases of infection COVID-19 among children in relation to the evolution of the pandemic. 07.06 - Paediatric respiratory epidemiology. Lausanne, Switzerland: European Respiratory Society (2022). 4305 p.

[39] Brainard J Grossi Sampedro CM Sweeting A Fordham R. Was alpha deadlier than wild-type COVID? Analysis in rural England. Infection. (2022) 50:1171–8. doi: 10.1007/s15010-022-01787-x 35247164 PMC8898029

[40] Li J-X Liao P-L Wei JC-C Hsu S-B Yeh C-J. A chronological review of COVID-19 case fatality rate and its secular trend and investigation of all-cause mortality and hospitalization during the Delta and omicron waves in the United States: a retrospective cohort study. Front Public Health. (2023) 11:1143650. doi: 10.3389/fpubh.2023.1143650 37799149 PMC10548482

[41] Tam NT Anh NT Tung TS Thach PN Dung NT Trang VD. Spatiotemporal evolution of SARS-CoV-2 alpha and Delta variants during large Nationwide outbreak of COVID-19, Vietnam, 2021. Emerg Infect Dis. (2023) 29:1002–1006. doi: 10.3201/eid2905.221787, PMID: 37015283 PMC10124647

[42] Esonova G Abdurakhimov A Ibragimova S Kurmaeva D Gulomov J Mirazimov D. Complete genome sequencing of SARS-CoV-2 strains that were circulating in Uzbekistan over the course of four pandemic waves. PLoS One. (2024) 19:e0298940. doi: 10.1371/journal.pone.0298940, PMID: 39561193 PMC11575833

[43] de Rioja VL Perramon-Malavez A Alonso S Andrés C Antón A Bordoy AE. Mathematical modeling of SARS-CoV-2 variant substitutions in European countries: transmission dynamics and epidemiological insights. Front Public Health. (2024) 12:1339267. doi: 10.3389/fpubh.2024.1339267 38855458 PMC11160439

[44] Chrysostomou A Vrancken B Haralambous C Alexandrou M Gregoriou I Ioannides M. Unraveling the dynamics of omicron (BA.1, BA.2, and BA.5) waves and emergence of the Deltacron variant: genomic epidemiology of the SARS-CoV-2 epidemic in Cyprus (Oct 2021–Oct 2022). Viruses. (2023) 15:1933. doi: 10.3390/v15091933 37766339 PMC10535466

[45] Atherstone CJ Guagliardo SAJ Hawksworth A O’Laughlin K Wong K Sloan ML. COVID-19 epidemiology during Delta variant dominance period in 45 high-income countries, 2020–2021. Emerg Infect Dis. (2023) 29:1757–1764. doi: 10.3201/eid2909.230142, PMID: 37494699 PMC10461680

[46] Cawley C Barsbay MÇ Djamangulova T Erdenebat B Cilović-Lagarija Š Fedorchenko V. The mortality burden related to COVID-19 in 2020 and 2021 - years of life lost and excess mortality in 13 countries and sub-national regions in southern and Eastern Europe, and Central Asia. Front Public Health. (2024) 12:1378229. doi: 10.3389/fpubh.2024.1378229, PMID: 38903591 PMC11187286

[47] Chief State Sanitary Doctor of the Republic of Kazakhstan. Resolution №36 of august 25, 2021: On sanitary and anti-epidemic measures to prevent coronavirus infection in educational institutions for the 2021–2022 academic year. Nur-Sultan, Kazakhstan: Ministry of Health of the Republic of Kazakhstan. (2021).

[48] Chief State Sanitary Doctor of the Republic of Kazakhstan. Resolution № 46 of October 20, 2021: On further implementation of measures to prevent coronavirus infection among the population of the Republic of Kazakhstan. Nur-Sultan, Kazakhstan: Ministry of Health of the Republic of Kazakhstan. (2021).

[49] World in Data is a project of Global Change Data Lab. COVID-19 data explorer. Vaccinations. Kazakhstan. Oxford, UK: Global Change Data Lab. (2024). Available online at: https://ourworldindata.org/explorers/covid?time=2021-01-31.2023-01-17&region=Asia&country=~KAZ&pickerSort=asc&pickerMetric=location&Metric=People+fully+vaccinated&Interval=Cumulative&Relative+to+population=true (Accessed May 5, 2025).

[50] Paton RS Overton CE Ward T. The rapid replacement of the SARS-CoV-2 Delta variant by omicron (B.1.1.529) in England. Sci Transl Med. (2022) 14:1–11. doi: 10.1126/scitranslmed.abo5395, PMID: 35503007 PMC9097877

[51] Allen H Tessier E Turner C Anderson C Blomquist P Simons D. Comparative transmission of SARS-CoV-2 omicron (B.1.1.529) and Delta (B.1.617.2) variants and the impact of vaccination: national cohort study, England. Epidemiol Infect. (2023) 151:e58. doi: 10.1017/S0950268823000420, PMID: 36938806 PMC10125873

[52] Nyberg T Ferguson NM Nash SG Webster HH Flaxman S Andrews N. Comparative analysis of the risks of hospitalisation and death associated with SARS-CoV-2 omicron (B.1.1.529) and delta (B.1.617.2) variants in England: a cohort study. Lancet. (2022) 399:1303–12. doi: 10.1016/S0140-6736(22)00462-7, PMID: 35305296 PMC8926413

[53] Torjesen I. Covid-19: omicron may be more transmissible than other variants and partly resistant to existing vaccines, scientists fear. BMJ. (2021) n2943. doi: 10.1136/bmj.n2943 34845008

[54] Elliott P Eales O Bodinier B Tang D Wang H Jonnerby J. Dynamics of a national omicron SARS-CoV-2 epidemic during January 2022 in England. Nat Commun. (2022) 13:4500. doi: 10.1038/s41467-022-32121-6 35922409 PMC9349208

[55] Glushkova N Ivankov A Trenina V Oshibayeva A Kalmatayeva Z Temirbekova Z. Post-conflict acute stress reactions in Kazakhstan in the aftermath of January 2022 unrests: a national survey. Heliyon. (2023) 9:e21065. doi: 10.1016/j.heliyon.2023.e21065 37964844 PMC10641126

[56] Elliott P Eales O Steyn N Tang D Bodinier B Wang H. Twin peaks: the omicron SARS-CoV-2 BA.1 and BA.2 epidemics in England. Science. (1979) 2022:376. doi: 10.1126/science.abq4411 PMC9161371 35608440

[57] Chadeau-Hyam M Tang D Eales O Bodinier B Wang H Jonnerby J. Omicron SARS-CoV-2 epidemic in England during February 2022: a series of cross-sectional community surveys. Lancet Regional Health Europe. (2022) 21:100462. doi: 10.1016/j.lanepe.2022.100462 35915784 PMC9330654

[58] Resolution of the Chief State Sanitary Doctor of Almaty July 04 2022 No. 6. On measures to prevent the spread of coronavirus infection in the territory of the city of Almaty. Almaty, Kazakhstan: Chief State Sanitary Doctor of Almaty. (2022).

[59] Carreño JM Wagner AL Monahan B Floda D Gonzalez-Reiche AS Tcheou J. SARS-CoV-2 serosurvey across multiple waves of the COVID-19 pandemic in new York City between 2020–2023. Cold Spring Harbor, NY: medRxiv (2023) 10.1038/s41467-024-50052-2 PMC11239669 38992013

